# Non-coding RNAs targeting NF-κB pathways in aquatic animals: A review

**DOI:** 10.3389/fimmu.2023.1091607

**Published:** 2023-02-06

**Authors:** Tanjun Zhao, Yang Zou, Hanyu Yan, Yaqing Chang, Yaoyao Zhan

**Affiliations:** ^1^ Key Laboratory of Mariculture & Stock Enhancement in North China’s Sea, Ministry of Agriculture and Rural Affairs, Dalian Ocean University, Dalian, China; ^2^ College of Life Science, Liaoning Normal University, Dalian, China

**Keywords:** nuclear factor-κB, microRNA, long noncoding RNA, circular RNA, ceRNA network

## Abstract

Nuclear factor-kappa B (NF-κB) pathways have a close relationship with many diseases, especially in terms of the regulation of inflammation and the immune response. Non-coding RNAs (ncRNAs) are a heterogeneous subset of endogenous RNAs that directly affect cellular function in the absence of proteins or peptide products; these include microRNAs (miRNAs), long noncoding RNAs (lncRNAs), circular RNAs (circRNAs), etc. Studies on the roles of ncRNAs in targeting the NF-κB pathways in aquatic animals are scarce. A few research studies have confirmed detailed regulatory mechanisms among ncRNAs and the NF-κB pathways in aquatic animals. This comprehensive review is presented concerning ncRNAs targeting the NF-κB pathway in aquatic animals and provides new insights into NF-κB pathways regulatory mechanisms of aquatic animals. The review discusses new possibilities for developing non-coding-RNA-based antiviral applications in fisheries.

## 1 Introduction

The nuclear factor-kappa B (NF-κB) pathways are well-known prototypical signaling pathways associated with inflammation, immune response, physiological stress, and disease occurrence in metazoans (1). According to differences in components and activation mechanisms, the NF-κB pathways can be further classified into canonical and non-canonical pathways (2). The canonical NF-κB pathway is currently recognized as a rapid and transient pathway closely related to pathogenesis of inflammatory diseases (1). This pathway includes NFκB1, p65 (also known as RelA), c-Rel, and activation of the signal-induced phosphorylation of IκB molecules by IκB kinases (IKKs) (3, 4). IKKs consist of two homologous catalytic subunits, IKKα and IKKβ (also known as IKK1 and IKK2), as well as a regulatory subunit IKKγ (also known as NF-κB essential modulator, NEMO) lacking catalytic capability. It has been demonstrated that IKKβ is essential for regulating canonical NF-κB activation with the support of the IKKγ subunit rather than the IKKα (4–6). In contrast to the canonical NF-κB pathway, the non-canonical NF-κB pathway is characterized as a slow and persistent pathway specifically associated with immune response and inflammatory diseases. The non-canonical NF-κB pathway includes NF-κB inducing kinase (NIK, also known as MAP3K14), IKKα, and RelB/p52 heterodimers (acting as transcription factors). The central event of non-canonical NF-κB pathway activation is NIK-IKKα axis-induced activation of RelB/p52 heterodimers.

Starting from the exploration of XrelA in developing embryos of the African clawed frog (*Xenopus laevis*) in 1994 (7), over the past 28 years both the canonical and the non-canonical NF-κB pathways have been extensively studied in various animals (7–11). In terms of aquatic animals, the immune regulatory functions of NF-κB pathways have been studied in amphibians, crustacean species, and teleost fishes, including in species such as the African clawed frog (7), Pacific white shrimp (*Litopenaeus vannamei*) (8), zebrafish (*Danio rerio*) (9, 10), and orange-spotted grouper (*Epinephelus coioides*) (11). However, most of the studies on NF-κB pathways in aquatic animals have focused on how the pathways regulate host immunity, while there is a lack of systematic summaries and analyses of the factors regulating the NF-κB pathways.

Non-coding RNAs (ncRNAs), a class of endogenous RNAs that are transcribed from DNA and affect cellular function through themselves rather than proteins or peptide products (12). ncRNAs can be classified into two main categories of small non-coding RNAs and long non-coding RNAs (lncRNAs) depending on their lengths. Small non-coding RNAs include small nuclear RNAs (snRNAs), microRNAs (miRNAs) and piwi-interacting RNAs (piRNAs), etc.; lncRNAs mainly include linear lncRNAs and circular RNAs (circRNAs) (12, 13). miRNAs are typically about 20–25 nucleotides (nt) in length and are generated from primary miRNAs (pri-miRNAs) (14). As regulatory molecules, miRNAs achieve their post-transcriptional regulation function through silencing or suppressing complexes leading to cleavage or translational downregulation of their target genes (14). lncRNAs are defined as transcripts with length greater than 200 nt and without evident protein coding function transcribed by RNA polymerase I, II or III (15). circular RNAs, as their name implies, have a circular structure which imparts additional stability to them against exonuclease cleavage. circRNAs can serve as transcriptional regulators, miRNA sponges, and as protein templates, decoys, scaffolds, and recruiters (13, 15). In recent years, increasing evidence has demonstrated that ncRNAs can regulate multiple physiological and pathological processes in aquatic animals such as teleost fishes (16, 17).

In this review, we focus on research advances concerning ncRNAs (mainly on miRNAs, linear lncRNAs and circRNAs) targeting the NF-κB pathways in aquatic animals such as teleost fishes and echinoderms (**Table 1**). Moreover, we will discuss the regulatory mechanisms of these ncRNAs involved in the NF-κB pathways and their potential as biomarkers or targets for early warning, control, and therapy of pathogen-induced inflammatory diseases in several aquatic animals.

**Table 1:** Non-coding RNAs (ncRNAs) involved in this review.

Categories	Subclasses	ncRNAs
Small non-coding RNAs	MicroRNAs (miRNAs)	miR-15a-5p
		miR-19a
		miR-21
		miR-21-1
		miR-21-3p
		miR-27c-3p
		miR-29a-3p
		miR-30c-3-3p
		miRn-115
		miR-122
		miR-133
		miR-142a-3p
		miR-144
		miR-146a
		miR-148
		miR-148-1-5p
		miR-181b-2
		miR-182-3p
		miR-200a-3p
		miR-203
		miR-210
		miR-214
		miR-216a
		miR-217
		miR-217-5p
		miR-2187
Small non-coding RNAs	miRNAs	miR-2187-3p
		miR-3570
		miR-8159-5p
Long non-coding RNAs (lncRNAs)	Linear lncRNAs	IRL
		NARL
		MARL
		AANCR
		MIR2187HG
	Circular RNAs (circRNAs)	CircDtx1
		CircBCL2L1
		CircPIKfyve
		CircRasGEF1B
		CircSamd4a

## 2 miRNAs targeting NF-κB pathways

### 2.1 miRNAs targeting the canonical NF-κB pathways

The canonical NF-κB pathway can be activated rapidly in both innate and adaptive immune cells by numerous signals through various receptors, including pattern-recognition receptors (PRRs), T-cell receptors (TCRs), B-cell receptors (BCRs), and proinflammatory cytokine receptors (18–20). In addition, the activation of the canonical NF-κB pathway also requires the involvement of specific adaptor molecules, ubiquitin ligases, and protein kinases (21, 22). In aquatic animals, the role of the PRR family in the canonical NF-κB pathway has been extensively investigated.

**Figure 1:**
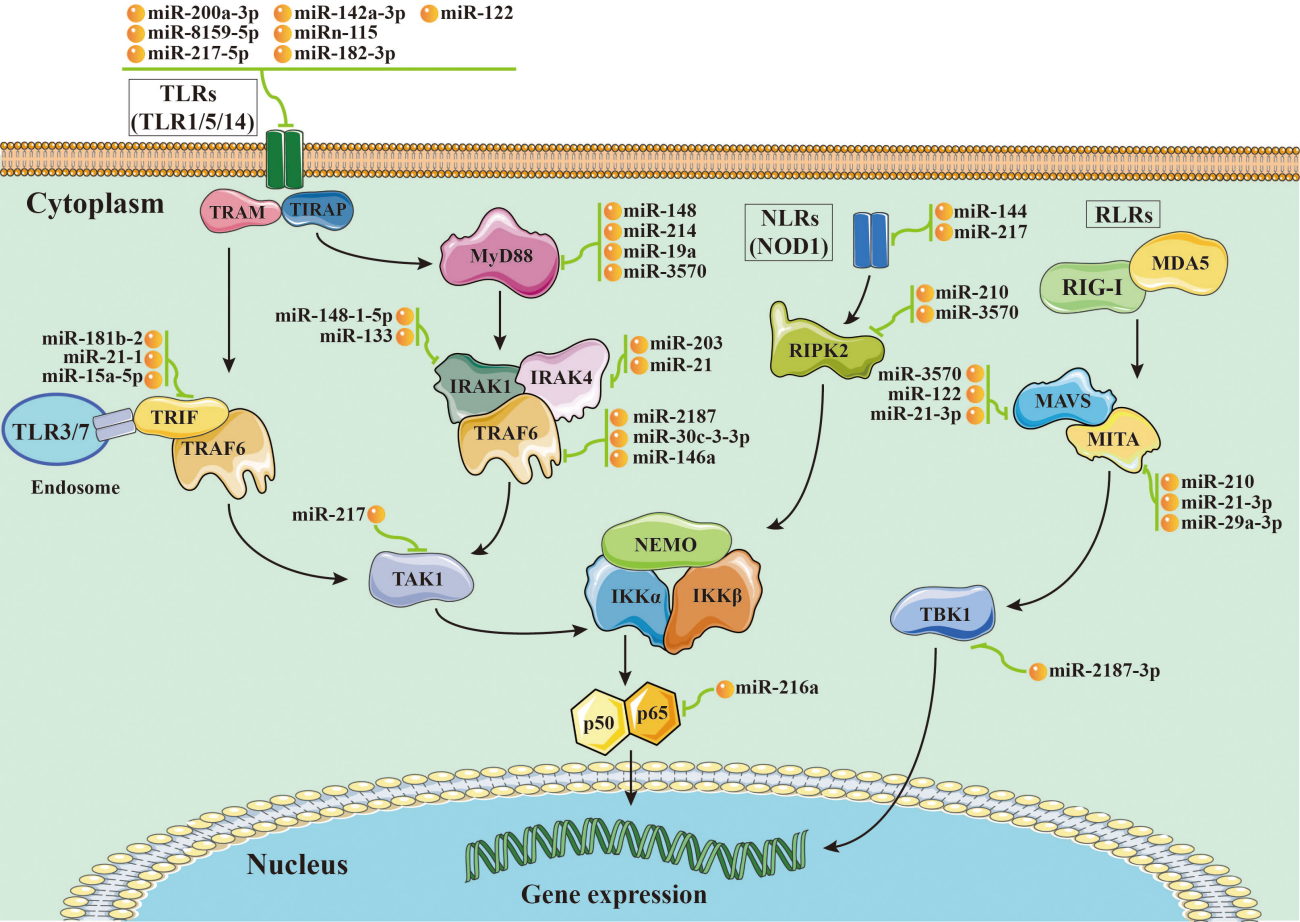
Schematic image of microRNA-mRNA interactions involved in nuclear factor-kappa B (NF-κB) pathways in aquatic animals.

It is accepted that most aquatic animals primarily rely on their innate immunity modulated by PRRs involved in signaling pathways due to their poorly developed adaptive immune systems (23, 24). In aquatic animals, the toll-like receptor (TLR), NOD-like receptor (NLR), and RIG-I-like receptor (RLR) signaling pathways are the most well characterized (23–28) (**Figure 1**). Moreover, key members of those signaling pathways play a variety of roles in organism growth and development (**Table 2**). Therefore, we will discuss miRNAs targeting TLR, NLR, and RLR signaling pathways in this section in detail.

**Table 2:** Summary of identified mRNAs associated with the nuclear factor-kappa B (NF-κB) pathway in aquatic animals.

mRNA	Function	Species	References
TLRs (TLR1/5/14)	Signaling ligand; pathogens recognition	*Miichthys miiuy;* *Ctenopharyngodon Idella;* *Epinephelus coioides*	(29–33)
TRIF	Signal transduction	*M. miiuy*	(34, 35)
MyD88	Signal transduction	*M. miiuy*	(17, 36–38)
IRAKs (IRAK1/4)	Kinases activity mediator	*M. miiuy;* *Apostichopus japonicus*	(39–43)
TRAF6	Intracellular signaling ligand	*M. miiuy;* *E. coioides*	(16, 43, 44)
TAK1	Upstream activator	*M. miiuy*	(45)
p65	Ligand	*M. miiuy*	(46)
NOD1	Intracellular receptor	*M. miiuy*	(47)
RIPK2	Downstream bridging protein	*M. miiuy*	(48)
MAVS	Mitochondrial signaling adapter	*M. miiuy*	(24–26)
MITA	Stimulator	*M. miiuy*	(49–51)
TBK1	Immune-related kinase	*M. miiuy*	(52)

#### 2.1.1 miRNAs targeting the TLR signaling pathways

The TLR pathways play a key role in the activation of innate immunity in eukaryotes by recognizing specific components of pathogens to detect pathogen invasion, which is known as a pathogen associated molecular pattern (PAMP) (53). The TLR pathways contain two axes, the “TLRs/TRIF-related adaptor molecule (TRAM)/Toll-IL-1 receptor domain-containing adaptor inducing interferon beta (TRIF)/TNFR-associated factor 6 (TRAF6)” axis and the “TLRs/Toll-IL-1 receptor adaptor protein (TIRAP)/Myeloid differentiation primary response gene 88 (MyD88)/IL-1 receptor-associated kinases (IRAKs, including IRAK1 and IRAK4)/TRAF6” axis. In aquatic animals, several key members of the TLR pathways have been identified, including cytoplasmic TLRs (TLR1, TLR5, and TLR14), TRIF, TRAF6, MyD88, IRAKs (IRAK1 and IRAK4), transforming growth factor beta-activated kinase 1 (TAK1), and p65 (**Figure 1**).

##### 2.1.1.1 miRNAs targeting TLRs

TLRs are important signaling recruiters, and they can be classified into cell membrane surface receptors (e.g., TLR1, TLR2, TLR4, TLR5, and TLR6) and intracellular receptors (e.g., TLR3, TLR7, TLR8, and TLR9) depending on their location in the cell (29, 53). In aquatic animals, the identified TLRs include TLR1, TLR5, and TLR14 in fish (30–33, 54), TLR1, TLR2, and TLR4 in the Pacific oyster (*Crassostrea gigas*) (55), TLR3 in sea cucumbers (*Apostichopus japonicus*) (56), TLR2 in the green mud crab (*Scylla paramamosain*), and TLR6, TLR7, TLR8, and TLR9 in Pacific white shrimp. In recent years, studies on miRNAs targeting TLRs have been conducted in fish.

**Figure 2:**
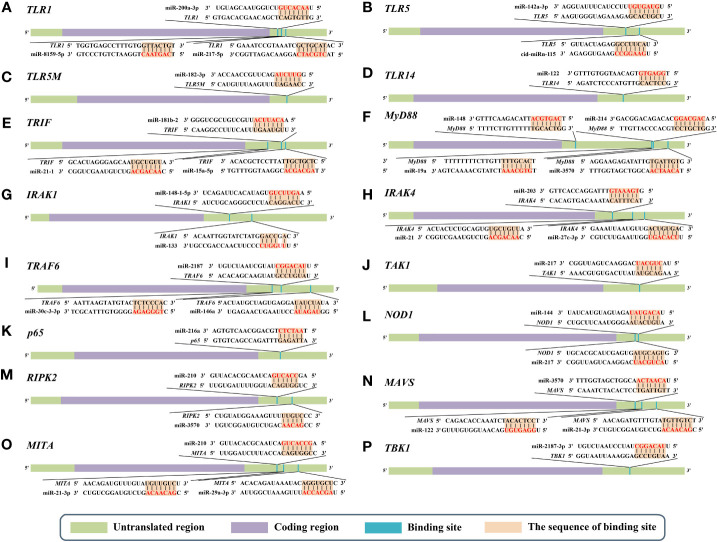
Predicted microRNA (miRNA) binding sites in the 3’ untranslated regions (UTRs) of different genes of nuclear factor-kappa B (NF-κB) pathways in aquatic animals. **(A)** Predicted binding sites in the 3’ UTR of *TLR1*. **(B)** Predicted binding sites in the 3’ UTR of *TLR5*. **(C)** Predicted binding sites in the 3’ UTR of *TLR5M* (the membrane form of TLR5). **(D)** Predicted binding sites in the 3’ UTR of *TLR14*. **(E)** Predicted binding sites in the 3’ UTR of *TRIF*. **(F)** Predicted binding sites in the 3’ UTR of *MyD88*. **(G)** Predicted binding sites in the 3’ UTR of *IRAK1*. **(H)** Predicted binding sites in the 3’ UTR of *IRAK4*. **(I)** Predicted binding sites in the 3’ UTR of *TRAF6*. **(J)** Predicted binding sites in the 3’ UTR of *TAK1*. **(K)** Predicted binding sites in the 3’ UTR of *p65*. **(L)** Predicted binding sites in the 3’ UTR of *NOD1*. **(M)** Predicted binding sites in the 3’ UTR of *RIPK2*. **(N)** Predicted binding sites in the 3’ UTR of *MAVS*. **(O)** Predicted binding sites in the 3’ UTR of *MITA*. **(P)** Predicted binding sites in the 3’ UTR of *TBK1*. The red markings represent seed regions.

miR-200a-3p, miR-8159-5p, and miR-217-5p are three known miRNAs with lengths of 22 nt, 23 nt, and 23 nt, respectively, and seed regions of “-AACACUG-” (miR-200a-3p), “-CAGTAAC-” (miR-8159-5p), and “-CTGCATC-” (miR-217-5p), respectively. Several studies have reported that miR-200a-3p, miR-8159-5p, and miR-217-5p can bind to the 3’ untranslated region (UTR) of the *TLR1* gene and exert negative regulation of post-transcriptional attenuation in the miiuy croaker (*Miichthys miiuy*) (**Figure 2A**). Pathogenic bacterial infection experiments indicated that the relative expression trends of miR-200a-3p, miR-8159-5p, and miR-217-5p were opposite to that of TLR1 in the spleen and leukocytes of miiuy croaker after infection with *Vibrio anguillarum* or stimulation with lipopolysaccharide (LPS), suggesting that miR-200a-3p, miR-8159-5p, and miR-217-5p may be involved in the immune response against pathogenic bacteria in fish by regulating the canonical NF-κB pathway *via* targeting TLR1 (30, 31).

miR-142a-3p (32), miRn-115 (32), and miR-182-3p (33) have been identified as miRNAs targeting TLR5 homologous genes in aquatic animals. miR-142a-3p, miRn-115, and miR-182-3p are 23 nt, 18 nt and 20 nt in length, respectively, with seed regions of “-GUAGUGU-” (miR-142a-3p), “-GAAGGCC-” (miRn-115), and “-GUUCUAG-” (miR-182-3p), respectively (**Figure 2B**). Dual luciferase reporter gene assays showed that both miR-142a-3p and miRn-115 could bind to the 3’ UTR of grass carp (*Ctenopharyngodon Idella*) *TLR5* and that they have a negative regulatory relationship (**Figure 2B**). Overexpression of miR-142a-3p or miRn-115 resulted in a significant decrease in the relative expression of the *TLR5* gene in the kidneys of grass carp infected with *Aeromonas hydrophila*, and this in turn suppressed the expression of downstream genes (e.g., interleukin-1β (*IL-1β*), interleukin-8 (*IL-8*), and tumor necrosis factor-*α* (*TNF-α*)) to avoid an excessive inflammatory response, suggesting that miR-142a-3p or miRn-115 could indirectly suppress the inflammatory response mediated by the canonical NF-κB pathway *via* negatively regulating the expression of *TLR5* homologous genes. miR-182-3p is another widely studied immunomodulatory-related miRNA in fish that has a length of 20 nt and a seed region of “-GUUCUAG-” (**Figure 2C**). As has been reported, miR-182-3p effectively inhibited the relative expression of *TLR5M* (the membrane form of TLR5) in the spleen cells of *E. coioides* during the immune response induced by flagellin of *Staphylococcus parapsilosis* (57) and consequently exerted organismal antibacterial immunity *via* inhibiting the activation of the TLR5M/MAPK/NF-κB pathway (32, 33).

miR-122 is a 22 nt miRNA that has a seed region of “-GGAGTG-”. miR-122 is involved in cell cycle regulation, cell differentiation, cell proliferation, and apoptosis processes in mammals (58–60). In aquatic animals, miR-122 is considered as a biomarker that contributes to the non-invasive diagnosis of liver injury in fish (61). TLR14 is a novel TLRs member that has been specifically identified in fish, including Japanese pufferfish (*Takifugu rubripes*) (GenBank accession No. AC156431.1), mandarin fish (*Siniperca chuatsi*) (GenBank accession No. MT594450.1), and miiuy croaker (*M. miiuy*) (GenBank accession No. KR709254.1) (54). A dual luciferase reporter gene assay indicated the presence of the seed region of miR-122 in the 3’ UTR of the *TLR14* gene (which belongs to the TLR2 subfamily) in miiuy croaker (**Figure 2D**). It was reported that the relative expression levels of miR-122 and TLR14 exhibited opposite trends in the spleen and macrophages of miiuy croaker after *V. anguillarum* infection and LPS stimulation, suggesting that miR-122 may affect the TLR14/NF-κB signaling cascade response by negatively regulating the relative expression of TLR14 (54).

##### 2.1.1.2 miRNAs targeting TRIF

TRIF, also known as TICAM-1 (62), is involved in immune response and disease regulation. Currently, *TRIF* homologs have been identified in a variety of aquatic organisms, including the Northeast Chinese lamprey (*Lethenteron morii*) (63), channel catfish (*Ictalurus punctatus*) (64), zebrafish (65), grass carp (66), orange-spotted grouper (67), and large yellow croaker (*Larimichthys crocea*) (34).

miRNAs targeting *TRIF* homologous genes in aquatic animals include miR-181b-2 (35), miR-21-1 (35), and miR-15a-5p (68) (**Figure 2E**). miR-181b-2, miR-21-1, and miR-15a-5p are 22 nt, 22 nt, and 21 nt in length, respectively, with highly similar seed regions of “-ACAUUCA-” (miR-181b-2), “-AACAGCA-” (miR-21-1), and “-AGCAGCA-” (miR-15a-5p), respectively. Dual luciferase reporter assays demonstrated that the seed regions of miR-181b-2, miR-21-1, and miR-15a-5p were all present in the 3’ UTR of *TRIF* in miiuy croaker (**Figure 2E**). The results of pathogenic infection experiments showed that the relative expression levels of miR-181b-2, miR-21-1, and miR-15a-5p in the macrophages and brain cells of miiuy croaker challenged by LPS stimulation and *V. anguillarum* or *Siniperca chuatsi* rhabdovirus (SCRV) infection showed opposite trends to those of TRIF (35). These observations suggested that miR-181b-2, miR-21-1, and miR-15a-5p could negatively regulate the expression of *TRIF* homologous genes and in turn regulate TRIF/NF-κB, ultimately inhibiting antibacterial and antiviral immune responses in fish (35).

##### 2.1.1.3 miRNAs targeting MyD88

MyD88, a key node in the canonical NF-κB signaling pathway, is also an important target for several miRNAs (3). To date, *MyD88* homologs have been identified in a variety of aquatic animals, including a marine gastropod (*Littorina littorea*) (69), a tropical sea cucumber (*Holothuria leucospilota*) (70), and a sea cucumber (*A. japonicus*) (36).

miR-148 (37), miR-214 (38), miR-19a (71), and miR-3570 (17) are known miRNAs that are 21 nt, 21 nt, 23 nt, and 22 nt in length, respectively, and contain seed regions “-CAGTGCA-” (miR-148), “-CAGCAGG-” (miR-214), “-GTGCAAA-” (miR-19a), and “-ACAATCA-” (miR-3570), respectively. These miRNAs have been validated to target and regulate the expression of *MyD88* homologs in different aquatic animals (**Figure 2F**). For example, dual luciferase assays have confirmed that the seed regions of miR-148, miR-214, miR-19a, and miR-3570 were simultaneously present in the 3’ UTR of *MyD88* in miiuy croaker (17, 37, 38, 71). Pathogenic bacterial infection experiments indicated that the relative expression trends of miR-148, miR-214, miR-19a, and miR-3570 were contrary to the relative expression levels of *MyD88* in the spleen, macrophages, and kidney of miiuy croaker after *Vibrio harveyi* infection and LPS stimulation, suggesting that miR-148, miR-214, miR-19a, and miR-3570 can negatively regulate the expression of *MyD88*. These observations also indicated that miR-148, miR-214, miR-19a, and miR-3570 may be involved in pathogenic bacteria-induced immune responses in fish through inhibiting the canonical NF-κB pathway *via* targeting *MyD88* homologs. Interestingly, it was reported that miR-19b (a member of the miR-19 family) with a seed region “-GUGCAAA-” significantly enhanced NF-κB activity in human HEK293 cells and mouse embryonic fibroblasts (72). Since there is no report concerning the role of miR-19b in aquatic animals, further study is necessary to clarify the regulation between miR-19b and the NF-κB pathways in aquatic animals.

##### 2.1.1.4 miRNAs targeting IRAKs

IRAK1 and IRAK4 are the only two members of the IRAK family with kinase activity (73–75), and IRAK1 can mediate NF-κB signaling by being recruited by MyD88, in turn leading to the production of inflammatory factors such as IL-8 and TNF-α (76). *IRAKs* have been identified in many aquatic organisms; for example, *IRAK1* homologs have been identified in red tailed shrimp (*Fenneropenaeus penicillatus*) (77) and Pacific white shrimp (78), and *IRAK4* homologs have been identified in an abalone (*Haliotis discus*) (GenBank accession No. KU351646.1), a thick shell mussel (*Mytilus coruscus*) (79), and a sea cucumber (*A. japonicus*) (39). Dual luciferase reporter assay data showed that 3’ UTRs of both *IRAK1* and *IRAK4* in aquatic animals have multiple miRNA target binding sites (**Figures 2G, H**). miR-148-1-5p (40), miR-133 (41), miR-203 (42), miR-21 (43), and miR-27c-3p (80) are known miRNAs with lengths of 23 nt, 23 nt, 22 nt, 22 nt and 21 nt, respectively, and the seed regions “-AGUUCUG-” (miR-148-1-5p), “-UUGGUCC-” (miR-133), “-TGAAATG-” (miR-203), “-AACAGCA-” (miR-21), and “-UCAGACU-” (miR-27c-3p), respectively. miR-148-1-5p and miR-133 can regulate the expression of *IRAK1*, and miR-203, miR-21, and miR-27c-3p regulate the expression of *IRAK4* (**Figure 2H**). Studies have shown that the expression levels of miR-148-1-5p in the brain cells of miiuy croaker and that of miR-133 in the coelomocytes of *A. japonicus* showed clear contradictory trends to those of *IRAK1* homologous genes after pathogenic bacterial infection as well as to the expression trends of miRNAs (miR-203, miR-21, and miR-27c-3p) and *IRAK4* in the liver, spleen, macrophages, kidney, and intestine cells of miiuy croaker. These observations indicated that miR-148-1-5p and miR-133 may act as negative regulators to inhibit the activation of the canonical NF-κB pathway by downregulating *IRAK1* homologous gene expression and that miR-203, miR-21, and miR-27c-3p could ultimately inhibit the antibacterial and antiviral immune effects of aquatic organisms *via* downregulation of *IRAK4* homologous gene expression.

##### 2.1.1.5 miRNAs targeting TRAF6

TRAF6 is an important intracellular multifunctional signaling molecule, and it is one of the most widely studied members of the TRAF family (81, 82). It has been reported that TRAF6 activated the IKK complex, in turn leading to the activation of NF-κB and the expression of inflammatory cytokines (83). Based on current research, TRAF6 has been identified in a variety of aquatic organisms, including Pacific white shrimp (69), Zhikong scallop (*Chlamys farreri*) (84), and Hong Kong oyster (*Crassostrea hongkongensis*) (GenBank accession No. MK799968.1).

miR-2187 (16), miR-30c-3-3p (44), and miR-146a (85) are 21 nt, 22 nt, and 23 nt in length, respectively, with seed regions “-UACAGGC-” (miR-2187), “-TGGGAGA-” (miR-30c-3-3p), and “-GUAGAUA-” (miR-146a), respectively. Dual luciferase reporter assays showed that *TRAF6* homologs in aquatic animals have binding sites (**Figure 2I**) on the 3’ UTR that exactly match the seed sequences of miR-2187, miR-30c-3-3p, and miR-146a. It has been reported that overexpression of miR-2187, miR-30c-3-3p, or miR-146a significantly suppressed the expression of TRAF6 in the liver and spleen cells of miiuy croaker infected with *V. anguillarum* and red-spotted grouper nervous necrosis virus (RGNNV), indicating a negative regulatory relationship between these miRNAs and TRAF6. In addition, overexpression of miR-2187, miR-30c-3-3p, or miR-146a also promoted the replication and occurrence of SCRV or RGNNV, suggesting that miR-2187, miR-30c-3-3p, or miR-146a could be induced and utilized by SCRV or RGNNV to be conducive to their infection and reproduction. Furthermore, it was also reported that miR-2187, miR-30c-3-3p, or miR-146a may possibly depress the expression of inflammatory factors (e.g., TNF-α, IL-8, or IL-1β) in an indirect way in the liver, kidney, and spleen cells of miiuy croaker or in the spleen of *E. coioides* after LPS stimulation or infection by RGNNV or SCRV (16, 44, 85). Combining all of the above observations, we conclude that miR-2187, miR-30c-3-3p, and miR-146a may enhance infection by SCRV and RGNNV through suppressing the NF-κB pathways and subsequently initiating inflammatory responses *via* targeting *TRAF6* homologs in fish.

##### 2.1.1.6 miRNAs targeting TAK1

TAK1 is a member of the MAPK kinase family, and it can be activated by TNF, LPS, and Epstein-Barr virus latent membrane protein 1 (LMP1) (86–89). Additionally, one of the most important roles of TAK1 is acting as an upstream activator of the NF-κB pathways (90). In aquatic animals, *TAK1* homologs have been identified in crustacean species and echinoderms such as the Pacific white shrimp (GenBank accession No. KU522004.1), a mud crab (*Scylla paramamamosain*) (GenBank accession No. MK319934.1), and a sea urchin (*Paracentrotus lividus*) (45).

miR-217 is the only known miRNA to date that may interact with TAK1 homologs in aquatic animals. This mRNA is 23 nt in length and has a “-CUGCAU-” seed region. Zhang et al. identified a binding site in the 3’ UTR of miiuy croaker TAK1 that exactly matched the seed sequence of miR-217 by using dual luciferase validation (91) (**Figure 2J**). In the Chinese mitten crab (*Eriocheir sinensis*), overexpression of miR-217 affects the replication of white spot syndrome virus (WSSV) and plays an active role in WSSV infection (92). Further *in vivo* and *in vitro* pathogenic infection experiments showed that the expression trends of miR-217 and *TAK1* were contrary in the spleen and macrophages of miiuy croaker, indicating that miR-217 may be involved in the antibacterial and antiviral immune responses of fish through suppressing the NF-κB pathways *via* targeting *TAK1* (91).

##### 2.1.1.7 miRNAs targeting p65

The p65 protein is located at the end of the canonical NF-κB pathway. The transcription of inflammatory cytokines is modulated by binding to p50 and forming a “p65/p50” dimer. The “p65/p50” dimer enters the nucleus and binds to the κB site in the promoter or enhancer of the target gene (93–97). In aquatic animals, *p65* homologous genes have been identified in teleost fishes such as zebrafish (9), olive flounder (*Paralichthys olivaceus*) (46), and common carp (*Cyprinus carpio*) (GenBank accession No. MN167531.1).

Currently, miR-216a is the only identified miRNA that can bind to the 3’ UTR of *p65* homologs in fish (98) (**Figure 2K**). miR-216a is 22 nt in length and has the seed region “-AATCTC-”. A study by Xu et al. (98) showed that the expression of miR-216a was significantly upregulated in the spleen and macrophages of miiuy croaker. Overexpression of miR-216a could also affect the expression of inflammatory cytokines such as TNF-α, IL-1β, interleukin-6 (IL-6), and IL-8. *In vivo* and *in vitro* experiments demonstrated that miR-216a could downregulate the activation of the NF-κB pathways by negatively regulating the expression of miiuy croaker p65 at the post-transcription level. All of the above-mentioned studies indicate that miR-216a could suppress excessive and prolonged inflammatory responses in the organism through negatively regulating the NF-κB pathways *via* directly targeting *p65* homologous genes in fish (98).

#### 2.1.2 miRNAs targeting the NLR signaling pathways

NLRs are important PRRs that sense bacterial products in the cytoplasm, and they play crucial roles in the recognition of bacterial or viral invasion in eukaryotic cells (47, 99). Key members of the NLR pathways include the NLR family members nucleotide oligomerization domain 1 and 2 (NOD1 and NOD2), receptor interacting serine/threonine kinase 2 (RIPK2), NEMO, IKKs (IKKα and IKKβ), and p50/p65. In aquatic animals, several members of the NLR pathway have been identified, including NOD1 (48, 100) and RIPK2 (101).

##### 2.1.2.1 miRNAs targeting NOD1

NOD1 is one of the most representative members of the NLR family, and it serves as an important intracellular receptor that effectively detects pathogenic components produced by various gram-negative bacteria in mammals (102, 103). NOD1 is involved in the antibacterial or antiviral-induced immune response by activating the NF-κB pathways and subsequent inflammatory responses (104, 105). In addition, NOD1 can also act as a receptor to enhance the immune response during viral infection (106). Currently, NOD1 has been identified in several aquatic organisms, including teleost fishes such as rainbow trout (*Oncorhynchus mykiss*) (GenBank accession No. KF484402.1), zebrafish (GenBank accession No. KC207831.1), mandarin fish (*S. chuatsi*) (GenBank accession No. KY974318.1), olive flounder (GenBank accession No. JF830013.1), and miiuy croaker (104).

miR-144 and miR-217 are 22 nt and 23 nt in length, respectively, with seed regions “-ACAGUAU-” (miR-144) and “-ACUGCAU-” (miR-217), respectively. Dual luciferase reporter assays have shown that *NOD1* homologs in aquatic animals have binding sites in the 3’ UTR that exactly match the seed sequences of miR-144 and miR-217 (**Figure 2L**). Overexpression of miR-144 and miR-217 significantly inhibited the expression of miiuy croaker *NOD1* at the post-transcription level in the spleen and macrophages of miiuy croaker infected with *V. harveyi* and stimulated by LPS, respectively, indicating a negative regulatory relationship between the above-mentioned miRNAs and *NOD1* (100). Taken together, the above-mentioned studies suggest critical roles for miR-144 and miR-217 that may involve inhibiting organismal and antibacterial immunity *via* negatively regulating the NF-κB pathways and subsequent inflammatory responses by suppressing *NOD1* expression (100).

##### 2.1.2.2 miRNAs targeting RIPK2

RIPK2 is a key factor involved in the pathogen-induced immune response (107–109). As the downstream bridging protein in the NLR signaling pathway, RIPK2 can influence cellular signaling and cytokine production induced by NOD1 (110). Currently, RIPK2 homologs have been identified in a number of aquatic animals, including cyclostome and teleost fishes such as the Reissner lamprey (*Lethenteron reissneri*) (111), goldfish (*Carassius auratus*) (GenBank accession No. KJ636470.1), and cyprinid fish (*Schizothorax prenanti*) (GenBank accession No. MW113673.1).

miR-210 and miR-3570 are two known miRNAs having regulatory relationships with *RIPK2* homologs (101). miR-210 and miR-3570 are both 22 nt in length, with seed regions “-CCACUG-” (miR-210) and “-CUGUU-” (miR-3570), respectively. Dual luciferase reporter assays revealed that both miR-210 and miR-3570 could precisely bind to the 3’ UTR of the *RIPK2* gene in miiuy croaker (101) (**Figure 2M**). Pathogenic bacterial infection experiments showed that in the spleen and macrophages of miiuy croaker infected by *V. harveyi* and subjected to LPS stimulation, the relative expression trends of miR-210 and miR-3570 were significantly in contrast to those of *RIPK2*. Considering all of the above results, we can conclude that miR-210 and miR-3570 may participate in the immune response against pathogenic bacterial or LPS stimulation in fish by negatively regulating the NF-κB pathways *via* targeting *RIPK2* (101).

#### 2.1.3 miRNAs targeting the RLR signaling pathways

RLRs play a key role in virus recognition and subsequent induction of antiviral immune responses (49, 112–114). Studies have demonstrated that RLRs can activate a signaling cascade that subsequently leads to the production of type-I interferons (IFNs) after efficient recognition of viral infection in the organism’s cytoplasm. The main members of the RLR pathway are retinoic acid-inducible gene I (RIG-I), melanoma differentiation-associated gene 5 (MDA5), mitochondrial antiviral signaling protein (MAVS), mediator of interferon regulatory factor 3 activation (MITA), and TANK-binding kinase 1 (TBK1). Key members of the RLR pathway identified in aquatic animals include MAVS (25–27), MITA (50–52), and TBK1 (115).

##### 2.1.3.1 miRNAs targeting MAVS

MAVS acts as a mitochondrial antiviral signaling adapter that bridges the gap between RIG-I and MDA5 sensing of viral infection and downstream signaling. Activation of MAVS leads to the rapid production of antiviral cytokines. *MAVS* homologs have been identified in teleost fishes such as zebrafish (116) and Atlantic salmon (*Salmo salar*) (117).

miR-3570 (25), miR-122 (26), and miR-21-3p (27) have lengths of 22 nt, 22 nt, and 22 nt, respectively, with seed regions “-ACAATCA-” (miR-3570), “-GGAGUG- “ (miR-122), and “-GACAACA-” (miR-21-3p) (**Figure 2N**). Dual luciferase reporter gene assays showed that miR-3570, miR-122, and miR-21-3p can bind to the 3’ UTR of the miiuy croaker *MAVS* gene and exert a negative regulatory effect on post-transcriptional attenuation. Pathogenic infection and poly(I:C) challenge experiments indicated that in macrophages, intestine cells, and kidney cells of miiuy croaker after SCRV infection, the relative expression of levels miR-3570, miR-122, or miR-21-3p were completely opposite to that of *MAVS*. Hence, the expression level promoted viral replication by inhibiting the production of IFNs and antiviral genes (e.g., *TNF-α*, *IL-1β*, and *Mx1*), ultimately inhibiting the organism’s antiviral response and promoting the replication of SCRV (25–27). Overall, these studies indicated that miR-3570, miR-122, and miR-21-3p acted as negative regulators in suppressing organismal immune and antiviral responses through depressing the NF-κB pathways *via* directly targeting MAVS (25–27).

##### 2.1.3.2 miRNAs targeting MITA

MITA is also known as a stimulator of interferon genes (STING) (118). As a crucial member of the RLR signaling pathway, MITA is involved in the regulation of signal transduction and the innate antiviral response in mammals (118). Compared to studies in mammals, there are relatively few studies related to MITA in aquatic animals.

miR-210, miR-21-3p, and miR-29a-3p are all 22 nt in length, with seed regions “-GCCACUG-” (miR-210), “-GACAACA-” (miR-21-3p), and “-AGCACCA-” (miR-29a-3p), respectively. Dual luciferase reporter assays demonstrated that *MITA* of miiuy croaker had binding sites (**Figure 2O**) in the 3’ UTR that precisely match the seed sequences of miR-210, miR-21-3p, and miR-29a-3p. Overexpression of miR-210, miR-21-3p, or miR-29a-3p suppressed the expression of MITA at both the mRNA and protein levels in the macrophages, intestinal cells and spleen cells of miiuy croaker after SCRV infection (50–52). Meanwhile, the expression of inflammatory cytokines (e.g., TNF-a, IL-6, and Mx1) was significantly inhibited. Taken together, these results suggest that miR-210, miR-21-3p, and miR-29a-3p may be involved in reducing cell proliferation, promoting viral replication, and inhibiting the organismal antiviral response by negatively regulating the NF-κB pathways *via* targeting *MITA* (50–52).

##### 2.1.3.3 miRNAs targeting TBK1

TBK1 is a vital immune-related kinase involved in the production of IFNs to prevent invasion by pathogenic microorganisms (119, 120). To date, *TBK1* homologous genes have been identified in several groups of aquatic animals, including mollusks and teleost fishes, in species such as marine gastropods (69), Pacific oyster (121), and zebrafish (122).

miR-2187-3p is the only miRNA that has a relationship with *TBK1* homologs in aquatic animals (115). This miRNA is 21 nt in length, with a seed region “-UACAGGCU-”. Dual luciferase reporter gene assays demonstrated that *TBK1* of miiuy croaker has a binding site in the 3’ UTR that completely matches the seed sequence of miR-2187-3p (**Figure 2P**) (115). In intestinal cells of miiuy croaker after SCRV infection, overexpression of miR-2187-3p downregulated the expression of TBK1 at both transcriptional and post-transcriptional levels. These observations suggested that miR-2187-3p may act as a negative regulator suppressing the organismal antiviral response through the NF-κB pathways *via* downregulating the relative expression of TBK1 (115).

**Figure 3:**
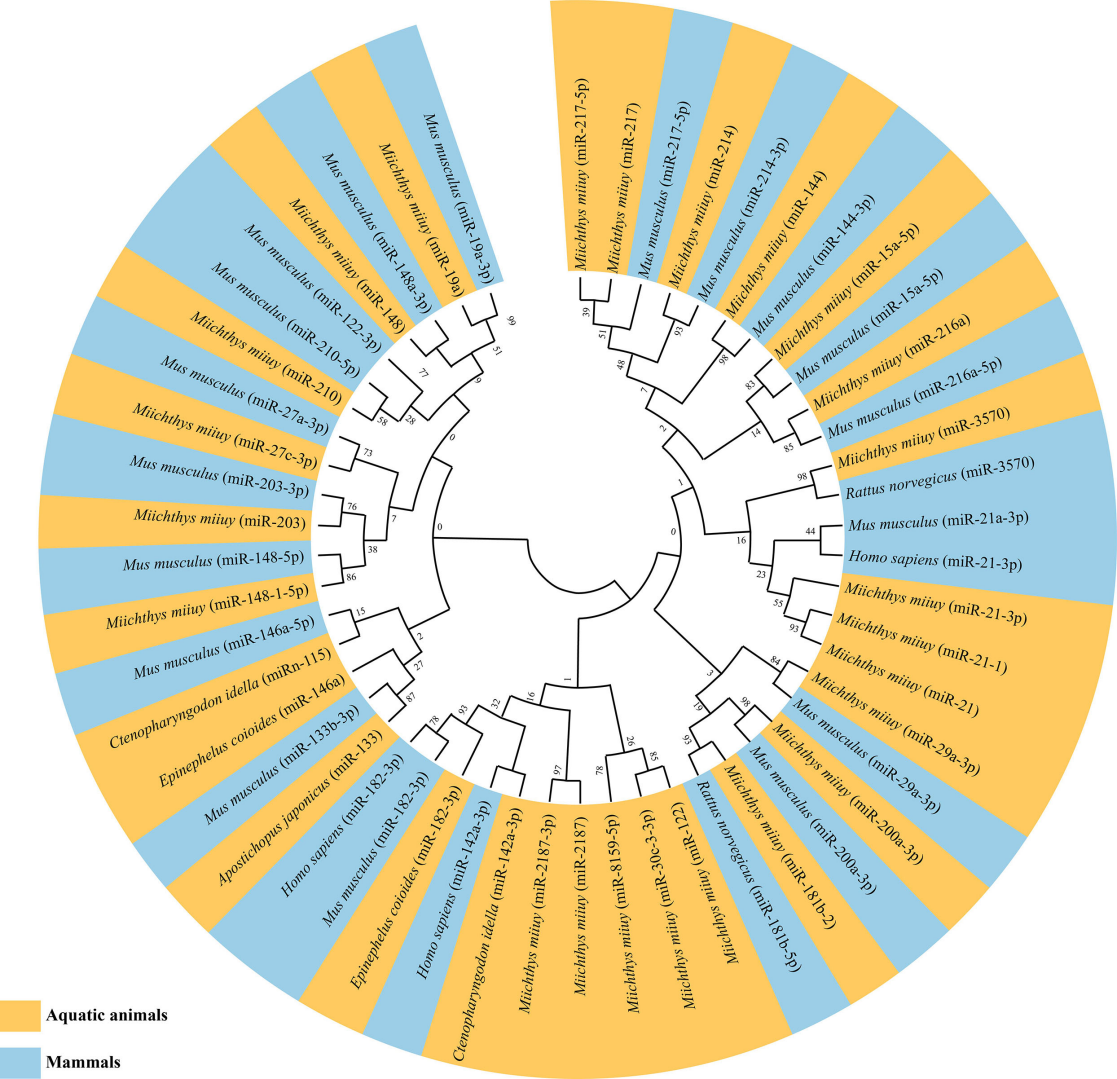
Maximum likelihood phylogenetic tree of identified microRNAs (miRNAs) regulating nuclear factor-kappa B (NF-κB) pathways in aquatic animals and mammals.

As mentioned above, miRNAs involved in NF-κB pathways have now been identified in a variety of aquatic organisms, especially in teleost fishes (123–154) (**Table 3**). Notably, most of these identified miRNAs have corresponding homologous sequences in mammals. Phylogenetic analysis data indicated a high conservation of examined miRNA homologous sequences in both aquatic animals and mammals (**Figure 3**), suggesting that these miRNAs might have similar regulatory mechanisms in different species. Furthermore, some miRNAs (miR-133, miR-21 and miR-210) have been identified both in vertebrate and non-vertebrate aquatic animals, whereas some have only been identified in teleost fishes (**Tables 3**, **4**). As the current relevant studies and evaluated aquatic species are still relatively few, whether there are differences in miRNAs regulating NF-κB pathways between vertebrate and non-vertebrate aquatic animals needs further exploration and justification.

**Table 3:** Summary of identified microRNAs (miRNAs) targeting genes of nuclear factor-kappa B (NF-κB) pathways in aquatic animals. “**-**”: negative regulation between miRNA and mRNA after experimental validation.

MiRNA	Species	Regulation (miRNA-mRNA)	Tissues		Target mRNA	References	
miR-200a-3p	*Miichthys miiuy*	**-**	Spleen; leukocytes		TLR1	(30)	
miR-8159-5p	*Salmo salar*; *M. miiuy*	**-**	Spleen; leukocytes			(31, 155) (31)	
miR-217-5p	*M. miiuy*	**-**	Spleen; leukocytes				
miR-142a-3p	*Ctenopharyngodon idella*; *Danio rerio*	**-**	Kidney		TLR5	(32, 129, 32)	
miRn-115	*C. idella*	**-**	Kidney				
miR-182-3p	*Epinephelus coioides*	**-**	Spleen			(33)
miR-122	*D. rerio*; *Oreochromis niloticus*; *M. miiuy*	**-**	Spleen; macrophages		TLR14	(54, 123, 124)	
miR-181b-2	*M. miiuy*	**-**	Macrophages; brain		TRIF	(35)	
miR-21-1	*M. miiuy*	**-**	Macrophages; brain				
miR-15a-5p	*Megalobrama amblycephala*; *M. miiuy*	**-**	Macrophages; brain			(68, 134)
miR-148	*M. miiuy*	**-**	Liver; spleen; macrophages; kidney		MyD88	(37)	
miR-214	*Siniperca chuatsi*; *M. miiuy*	**-**	Liver; macrophages			(38, 150)
miR-19a	*D. rerio*; Hybrid tilapia (*Oreochromis aureus*♂×*O. niloticus*♀); *Cyprinus carpio*; *Gadus morhua*; *M. miiuy*	**-**	macrophages			(71, 135–138)
miR-3570	*M. miiuy*	**-**	Liver; spleen; macrophages; kidney		MyD88	(17)	
miR-148-1-5p	*M. miiuy*	**-**	brain		IRAK1	(40)	
miR-133	*Oncorhynchus mykiss*; *Clarias magur*; *Pinctada martensii*; *Apostichopus japonicus*	**-**	coelomocytes			(41, 125–127)
miR-203	*D. rerio*; *C. carpio*; *Gobiocypris rarus*; *M. miiuy*	**-**	Liver; spleen; macrophages; kidney		IRAK4	(42, 139–141)	
miR-21	*Nothobranchius furzeri*; *O. niloticus*; *C. idella*; *D. rerio*; *Caligus rogercresseyi*; *M. miiuy*	**-**	Liver; spleen; macrophages			(43, 142–146)
miR-27c-3p	*M. miiuy*	**-**	Intestine			(80)
miR-2187	*M. miiuy*	**-**	Liver; spleen		TRAF6	(16)	
miR-30c-3-3p	*M. miiuy*	**-**	Kidney			(82)
miR-146a	*Siganus canaliculatus*; *E. coioides*; *S. chuatsi*;	**-**	Spleen			(85, 131, 133)
miR-217	*C. carpio*; *D. rerio*; *C. magu*r; *M. miiuy*	**-**	Spleen; macrophages		TAK1	(91, 123, 125, 153)
miR-216a	*D. rerio*; *C. carpio*; *M. miiuy*	**-**	Spleen; macrophages		p65	(98, 151, 152)	
miR-144	*D. rerio*; *M. miiuy*	**-**	Spleen; macrophages		NOD1	(100, 100, 123, 125, 130, 153)	
miR-217	*C. carpio*; *D. rerio*; *C. magur*; *M. miiuy*	**-**	Spleen; macrophages				
miR-210	*O. mykiss*; *Oryzias melastigma*, *Litopenaeus vannamei*; *M. miiuy*	**-**	Spleen; macrophages		RIPK2	(101, 147–149)	
miR-3570	*M. miiuy*	**-**	Spleen; macrophages				
miR-3570	*M. miiuy*	**-**	Macrophages		MAVS	(25)	
miR-122	*D. rerio*; *O. niloticus*; *M. miiuy*	**-**	Intestine			(26, 123, 124)
miR-21-3p	*M. miiuy*	**-**	Kidney; intestine		MAVS	(27)	
miR-210	*O. melastigma*; *L. vanname*i; *M. miiuy*	**-**	Macrophages		MITA	(50, 147–149)	
miR-21-3p	*M. miiuy*	**-**	Intestine			(51)
miR-29a-3p	*O. mykiss*; *M. miiuy*	**-**	Spleen; intestine			(52, 156)
miR-2187-3p	*M. miiuy*	**-**	Intestine		TBK1	(115)	

**Table 4:** microRNAs (miRNAs) sequences used for phylogenetic analysis.

MiRNA	Sequence	Taxon	Species
miR-15a-5p	TAGCAGCACGGAATGGTTTGT	Teleost fishes	*Miichthys miiuy*
miR-15a-5p	UAGCAGCACAUAAUGGUUUGUG	Mammals	*Mus musculus*
miR-19a	TGTGCAAATCTATGCAAAACTGA	Teleost fishes	*M. miiuy*
miR-19a-3p	UGUGCAAAUCUAUGCAAAACUGA	Mammals	*M. musculus*
miR-21	CAACAGCAGUCUGUAAGCUGGC	Teleost fishes	*M. miiuy*
miR-21-1	CAACAGCAGUCUGUAAGCUGGC	Teleost fishes	*M. miiuy*
miR-21-3p	CGACAACAGUCUGUAGGCUGUC	Teleost fishes	*M. miiuy*
miR-21a-3p	CAACAGCAGUCGAUGGGCUGUC	Mammals	*M.musculus*
miR-21-3p	CAACACCAGUCGAUGGGCUGU	Mammals	*Homo sapiens*
miR-27a-3p	UUCACAGUGGCUAAGUUCCGC	Mammals	*M.musculus*
miR-27c-3p	UUCACAGUGGUUAAGUUCUGC	Teleost fishes	*M. miiuy*
miR-29a-3p	UAGCACCAUUUGAAAUCGGUUA	Teleost fishes	*M. miiuy*
miR-29a-3p	UAGCACCAUCUGAAAUCGGUUA	Mammals	*M.musculus*
miR-30c-3-3p	CTGGGAGAGGGGTGTTTACGCT	Teleost fishes	*M. miiuy*
miRn-115	UGAAGGCCGAAGUGGAGA	Teleost fishes	*Ctenopharyngodon idella*
miR-122	TGGAGTGTGACAATGGTGTTTG	Teleost fishes	*M. miiuy*
miR-122-3p	AAACGCCAUUAUCACACUAAAU	Mammals	*M.musculus*
miR-133	UUUGGUCCCCUUCAACCAGCCGU	Echinoderms	*Apostichopus japonicus*
miR-133b-3p	UUUGGUCCCCUUCAACCAGCUA	Mammals	*M.musculus*
miR-142a-3p	UGUAGUGUUUCCUACUUUAUGGA	Teleost fishes	*C. idella*
miR-142a-3p	UGUAGUGUUUCCUACUUUAUGGA	Mammals	*H. sapiens*
miR-144	UACAGUAUAGAUGAUGUACUAU	Teleost fishes	*M. miiuy*
miR-144-3p	UACAGUAUAGAUGAUGUACU	Mammals	*M.musculus*
miR-146a	GGUAGAUACCUUAAGUCAAGAGU	Teleost fishes	*E. coioides*
miR-146a-5p	UGAGAACUGAAUUCCAUGGGUU	Mammals	*M.musculus*
miR-148	TCAGTGCATTACAGAACTTTG	Teleost fishes	*M. miiuy*
miR-148-1-5p	AAGUUCUGUGAUACACUUAGACU	Teleost fishes	*M. miiuy*
miR-148a-3p	UCAGUGCACUACAGAACUUUGU	Mammals	*M.musculus*
miR-148-5p	AAAGUUCUGAGACACUCCGACU	Mammals	*M.musculus*
miR-181b-2	AACAUUCAUUGCUGUCGCUGGG	Teleost fishes	*M. miiuy*
miR-181b-5p	AACAUUCAUUGCUGUCGGUGGGU	Mammals	*Rattus norvegicus*
miR-182-3p	GGUUCUAGACUUGCCAACCA	Teleost fishes	*E. coioides*
miR-182-3p	UGGUUCUAGACUUGCCAACUA	Mammals	*H. sapiens*
miR-182-3p	GUGGUUCUAGACUUGCCAACU	Mammals	*M.musculus*
miR-200a-3p	UAACACUGUCUGGUAACGAUGU	Teleost fishes	*M. miiuy*
miR-200a-3p	UAACACUGUCUGGUAACGAUGU	Mammals	*M.musculus*
miR-203	GTGAAATGTTTAGGACCACTTG	Teleost fishes	*M. miiuy*
miR-203-3p	GUGAAAUGUUUAGGACCACUAG	Mammals	*M.musculus*
miR-210	AGCCACUGACUAACGCACAUUG	Teleost fishes	*M. miiuy*
miR-210-5p	AGCCACUGCCCACCGCACACUG	Mammals	*M.musculus*
miR-214	ACAGCAGGCACAGACAGGCAG	Teleost fishes	*M. miiuy*
miR-214-3p	ACAGCAGGCACAGACAGGCAGU	Mammals	*M.musculus*
miR-216a	TAATCTCTGCAGGCAACTGTGA	Teleost fishes	*M. miiuy*
miR-216a-5p	UAAUCUCAGCUGGCAACUGUGA	Mammals	*M.musculus*
miR-217	UACUGCAUCAGGAACUGAUUGGC	Teleost fishes	*M. miiuy*
miR-217-5p	TACTGCATCAGGAACAGATTGGC	Teleost fishes	*M. miiuy*
miR-217-5p	UACUGCAUCAGGAACUGACUGGA	Mammals	*M.musculus*
miR-2187	UUACAGGCUAUGCUAAUCUGU	Teleost fishes	*M. miiuy*
miR-2187-3p	UUACAGGCUAUCCUAAUCUGU	Teleost fishes	*M. miiuy*
miR-3570	TACAATCAACGGTCGATGGTTT	Teleost fishes	*M. miiuy*
miR-3570	GGUACAAUCAACGGUCGAUGGU	Mammals	*R. norvegicus*
miR-8159-5p	TCAGTAACTGGAATCTGTCCCTG	Teleost fishes	*M. miiuy*

## 3 ceRNAs involved in the NF-κB pathways

**Figure 4:**
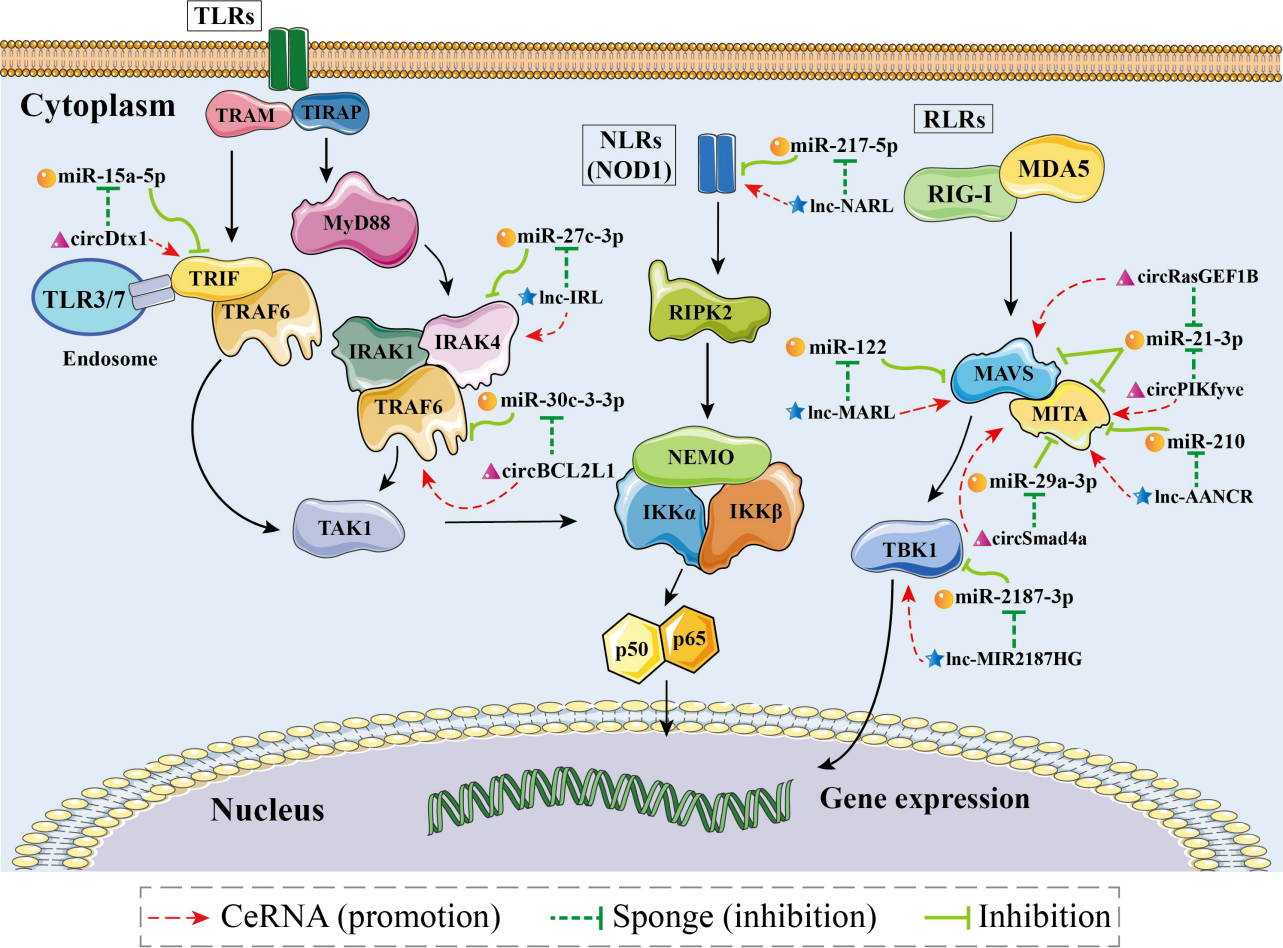
Schematic image of identified competing endogenous RNA (ceRNA) networks associated with nuclear factor-kappa B (NF-κB) pathways in aquatic animals.

Competing endogenous RNA (ceRNA) is defined as RNAs such as linear lncRNAs, circRNA and even mRNAs with the miRNA response element (MRE) that can bind competitively to miRNAs and make them nonfunctional (157). As a novel molecular mechanism in RNA interactions, the hypothesis of ceRNA action was first proposed in 2011 (157). It has been extensively documented that ceRNA can act as a sponge to attract and isolate miRNAs, thereby blocking the effects of miRNAs on their target genes (155–159). According to different ncRNA components, ceRNA networks can be classified into four types: “lncRNA-miRNA-mRNA”, “mRNA-miRNA-mRNA”, “circRNA-miRNA-mRNA”, and “pseudogene-miRNA-mRNA” (155, 159, 160). Several studies have reported that in aquatic animals, ceRNA regulatory networks formed by the participation of ncRNAs (lncRNAs, circRNAs, and miRNAs) play important roles in the innate immune response of the organism, especially in the NF-κB pathways (**Figure 4**, **Table 5**).

**Table 5:** Summary of identified competing endogenous RNAs (ceRNAs) associated with genes of the nuclear factor-kappa B (NF-κB) pathways in aquatic animals.

CeRNA network type	CeRNA	Shared miRNA	Target mRNA	Species	References
LncRNA-miRNA-mRNA	IRL	miR-27c-3p	IRAK4	*Miichthys miiuy*	(43)
	NARL	miR-217-5p	NOD1		(100)
	MARL	miR-122	MAVS		(25)
	AANCR	miR-210	MITA		(129)
	MIR2187HG	miR-2187-3p	TBK1		(52)
CircRNA-miRNA-mRNA	CircDtx1	miR-15a-5p	TRIF		(35)
	CircBCL2L1	miR-30c-3-3p	TRAF6		(84)
	CircPIKfyve	miR-21-3p	MAVS		(26)
	CircRasGEF1B	miR-21-3p	MITA		(50)
	CircSamd4a	miR-29a-3p			(51)

### 3.1 “lncRNA-miRNA-mRNA” networks regulating the NF-κB pathways

lncRNAs can achieve regulatory effects by interacting with other ncRNAs, mRNAs, proteins, and genomic DNA. lncRNAs play a crucial role in the regulation of gene expression during transcription and translation, as well as at the epigenetic level by performing different functions, including signaling, guidance, decoy, and sponge roles. Therefore, lncRNAs are considered to be pleiotropic and as “master regulators” of the genome (161). Among aquatic animals, lncRNAs have mostly been studied in teleost fishes. In this section, we discuss the “lncRNA-miRNA-mRNA” ceRNA networks that have been adequately identified in aquatic animals, including “IRAK4-related lncRNA (IRL)/miR-27c-3p/IRAK4” (80), “NOD1 antibacterial and antiviral-related lncRNA (NARL)/miR-217-5p/NOD1” (48), “MAVS antiviral-related lncRNA (MARL)/miR-122/MAVS” (26), “antiviral-associated lncRNA (AANCR)/miR-210/MITA” (162), and “MIR2187HG/miR-2187-3p/TBK1” (115).

#### 3.1.1 “lncRNA-miRNA-mRNA” networks regulating the TLR signaling pathway

**Figure 5:**
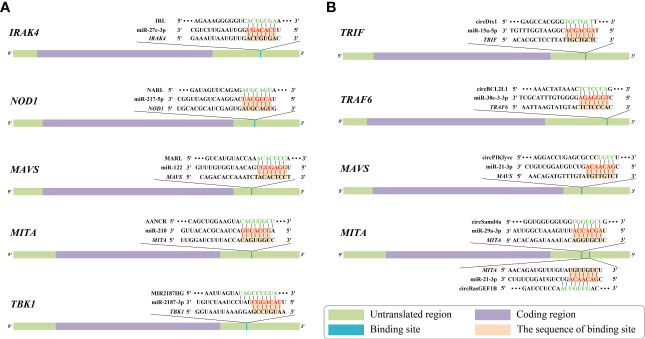
Predicted binding sites of identified competing endogenous RNAs (ceRNAs) associated with genes of nuclear factor-kappa B (NF-κB) pathways in aquatic animals. **(A)** Predicted binding sites of “long non-coding RNA (lncRNA)-microRNA (miRNA)-mRNA” ceRNA networks. **(B)** Predicted binding sites of “circular RNA (circRNA)-miRNA-mRNA” ceRNA networks. The red markings represent seed regions.

The “IRL/miR-27c-3p/IRAK4” network is a key ceRNA network related to immune defense in aquatic organisms (80). Dual luciferase reporter assays have demonstrated that IRL can bind to the seed sequence of miR-27c-3p (**Figure 5A**). Thus, IRL was able to activate and upregulate the expression of *IRAK4* by functioning as a ceRNA, while miR-27c-3p acted as a repressor to *IRAK4*. Under the conditions of knockdown of IRL and overexpression of miR-27c-3p after LPS stimulation, the expression of *IRAK4* and subsequent inflammatory factors was significantly suppressed, indicating that miR-27c-3p and IRL play important regulatory roles in the inflammatory response of miiuy croaker. IRAK4 has been repeatedly shown to be an important factor in the TLR-dependent immune response; it has a role in promoting cell proliferation, while inhibition of its expression promotes apoptosis (163–167). It has been reported that IRAK4 can induce innate antimicrobial responses through the NF-κB pathways, thereby activating inflammatory factors in response to external stimuli in fish (168–170). Collectively, IRL may promote organismal immune responses through upregulating the NF-κB pathways *via* sponging miR-27c-3p and acting as a ceRNA to IRAK4, while miR-27c-3p acts conversely (80). These studies suggested that the “IRL/miR-27-3p/IRAK4” network plays an important role in the inflammatory response, and the results contribute to further understanding of aquatic biological immunology, as well as to that of disease control mechanisms (80).

#### 3.1.2 "lncRNA-miRNA-mRNA" networks regulating the NLR signaling pathways

The “NARL/miR-217-5p/NOD1” network is the only known ceRNA network to date that regulates the NLR signaling pathway in aquatic animals (48). Zheng et al. demonstrated a binding site between NARL and miR-217-5p by using dual luciferase validation (**Figure 5A**). In this process, the expression levels of NARL and *NOD1* increased, while those of miR-217-5p decreased; the functional attenuation of miR-217-5p thus resulted in the enhancement of the immune response and the NF-κB pathways in miiuy croaker. Specifically, NARL competitively binds to miR-217-5p in order to counteract its inhibition of *NOD1* by exerting a sponge effect after LPS stimulation or SCRV infection, indicating that miR-217-5p and NARL have competitive relationships with *MmiNOD1*. It is generally acknowledged that NOD1 activates the NF-κB pathways to promote host production of multiple inflammatory cytokines to resist bacterial invasion in fish. Moreover, NOD1 can also act as an RNA virus receptor to enhance the immune response during viral infection (106). Considering all of this evidence, it seems that miR-217-5p/*NOD1* can be further regulated by NARL to achieve more precise immune homeostasis (48). The above-mentioned studies revealed that miR-217-5p and NARL regulate NOD1 and subsequent immune defense processes of an organism through their mutually associated ceRNA activities. The “NARL/miR-217-5p/NOD1” network could be a key node for further understanding the molecular regulatory mechanisms of immune responses in aquatic organisms (48).

#### 3.1.3 “lncRNA-miRNA-mRNA” networks regulating the RLR signaling pathways

Currently, three “lncRNA-miRNA-mRNA” ceRNA networks have been identified as regulating the RLR signaling pathway in aquatic animals, namely “MARL/miR-122/MAVS” (25), “AANCR/miR-210/MITA” (162), and “MIR2187HG/miR-2187-3p/TBK1” (115).

“MARL/miR-122/MAVS” was identified as a ceRNA network in aquatic animals. Dual luciferase reporter assays verified that MARL acts as a sponge by competitively binding to miR-122 (**Figure 5A**), thereby interacting with miR-122 and acting as a ceRNA of MAVS and in turn promoting the expression at both the mRNA level and the protein level, thereby enhancing the antiviral signaling pathway (26). Further functional experiments demonstrated that the expression of MAVS decreased after knockdown of MARL. In contrast, overexpression of miR-122 can suppress the activation of the NF-κB pathways and downstream inflammatory response, thereby helping the virus to evade the host antiviral response *via* inhibiting the expression of *MAVS*. The host signaling protein MAVS is critical in driving the antiviral innate immune response to RNA virus infection (171). MARL could counteract the upregulatory effect of miR-122 on SCRV replication, thus maintaining the stability of the antiviral response and ensuring an appropriate inflammatory response. Taken together, these observations indicated that the “MARL/miR-122/MAVS” network may play an important role in aquatic animals’ immune responses, especially inflammatory responses, as well as in the NF-κB signaling pathways (26).

The “AANCR/miR-210/MITA” network, a vital ceRNA network regulating the RLR signaling pathway related to MITA, was identified in miiuy croaker (162). Dual luciferase report assays demonstrated that AANCR has an intact binding site with miR-210 (**Figure 5A**). It has been reported that AANCR could act as a sponge for miR-210 that subsequently indirectly regulates the NF-κB pathways. This molecular regulatory mechanism of the ceRNA network contributes to the recognition and elimination of viruses by the host immune system (162).

The “MIR2187HG/miR-2187-3p/TBK1” network is a key ceRNA network related to antibacterial and antiviral responses in aquatic animals. TBK1 can promote canonical activation of NF-κB and interferon regulatory factor 3 (IRF3) to accelerate proinflammatory gene transcription (172). It has been reported that overexpression of MIR2187HG could inhibit the expression of miR-2187-3p and upregulate that of miiuy croaker *TBK1* in a manner that competitively binds to miR-2187-3p (**Figure 5A**), consequently restoring the immune response mediated by the NF-κB pathways (115). MIR2187HG may promote organismal immune responses through upregulating the NF-κB pathways *via* sponging miR-2187-3p and being a ceRNA indirectly targeting TBK1 (115). Taken together, we can hypothesize that the “MIR2187HG/miR-2187-3p/TBK1” network could be a key entry point for further understanding the molecular regulatory mechanisms of immune responses in aquatic organisms (115).

### 3.2 “circRNA-miRNA-mRNA” networks regulating the NF-κB pathways

CircRNAs are novel ncRNAs with a stable structure of covalently closed continuous loops (173). Current data show that circRNAs can act as miRNA sponges and subsequently inactivate the post-transcriptional attenuation functions of corresponding miRNAs (174, 175). To date, most studies concerning circRNAs have focused on human diseases; studies on circRNAs’ targets (such as mRNA and miRNA) are largely lacking. In this section, we discuss five “circRNA-miRNA-mRNA” networks associated with NF-κB pathways, namely “circDtx1/miR-15a-5p/TRIF” (68), “circBCL2L1/miR-30c-3-3p/TRAF6” (44), “circPIKfyve/miR-21-3p/MAVS” (27), “circRasGEF1B/miR-21-3p/MITA” (51), and “circSamd4a/miR-29a-3p/MITA” (52).

#### 3.2.1 "circRNA-miRNA-mRNA" networks regulating the TLR signaling pathways

The “CircDtx1/miR-15a-5p/TRIF” network (68) and the “circBCL2L1/miR-30c-3-3p/TRAF6” network (44) are two identified “circRNA/miRNA/mRNA” ceRNA networks regulating the TLR signaling pathway in aquatic animals.

The “circDtx1/miR-15a-5p/TRIF” network has been fully identified as being involved in the TLR signaling pathway in aquatic animals. Dual luciferase reporter assays have demonstrated that circDtx1 can bind to the seed sequence of miR-15a-5p that in turn inhibits the miR-15a-5p that had an intact binding site on the 3’ UTR of *TRIF* (**Figure 5B**). CircDtx1 acts as a ceRNA that upregulates the expression of TRIF at both the mRNA level and protein level by sponging miR-15a-5p, thereby promoting the organismal antiviral response *via* the NF-κB pathways (68). Overexpression of circDtx1 led to a decrease in the expression of miR-15a-5p, indicating a negative correlation. TRIF is a key member of the NF-κB pathways, and it has an important role in activating NF-κB signaling. These observations indicated that circDtx1 could function as a sponge of miR-15a-5p that forms the “circDtx1/miR-15a-5p/TRIF” ceRNA network to suppress viral replication and enhance immunological activity. These observations provide new insights into the role of circRNAs in host antiviral immunity (68).

Additionally, it was reported that circBCL2L1 could function as a molecular sponge of miR-30c-3-3p in the “circBCL2L1/miR-30c-3-3p/TRAF6” ceRNA network (44). As stated in previous studies, there is a strong correlation between TRAF6 and the innate immune response (83, 176). Dual luciferase reporter assays showed that circBCL2L1 had an intact binding site with miR-30c-3-3p (**Figure 5B**). Further experiments confirmed that overexpression of circBCL2L1 could promote organismal antibacterial and antiviral responses through enhancing the NF-κB pathways *via* competitively binding to miR-30c-3-3p and upregulating the relative expression of TRAF6. In addition, it was reported that circBCL2L1 could restore the attenuated immune response induced by miR-30c-3-3p and in turn maintain the stability of the immune response, thereby ensuring an appropriate inflammatory response.

#### 3.2.2 “circRNA-miRNA-mRNA” networks regulating the RLR signaling pathways

Currently, there are three “circRNA-miRNA-mRNA” networks, the “circPIKfyve/miR-21-3p/MAVS” network (27), the “circRasGEF1B/miR-21-3p/MITA” network (51), and the “circSamd4a/miR-29a-3p/MITA” network (52), that have been identified as novel regulatory mechanisms of the RLR signaling pathway in aquatic animals.

Su et al. indicated that the “circPIKfyve/miR-21-3p/MAVS” network is a crucial ceRNA network associated with the RLR signaling pathway in aquatic organisms (27). Dual luciferase reporter assays demonstrated that circPIKfyve could target and bind to miR-21-3p (**Figure 5B**). Consequently, circPIKfyve could activate and upregulate the expression of *MAVS* by functioning as a ceRNA, while miR-21-3p acted as a repressor of *MAVS*. The expression levels of *MAVS* and inflammatory cytokines were remarkably reduced as a result of miR-21-3p overexpression and IRL knockdown after LPS stimulation. To date, studies have confirmed that MAVS is a key member of the RLR pathway-related innate antiviral immune response and NF-κB pathways whose activation leads to rapid production of antiviral cytokines (114, 177). miR-21-3p is a novel miRNA that directly targets MAVS, negatively regulates the relative expression of MAVS, and inhibits the antimicrobial response it mediates. Overall, the above observations suggest that circPIKfyve may promote the organismal immune response by upregulating the NF-κB pathways *via* sponging miR-21-3p and being a ceRNA to MAVS. This observation also highlights the finding that the “circPIKfyve/miR-21-3p/MAVS” network plays an important role in the inflammatory response as well as NF-κB pathways in aquatic animals.

It has been shown that the “circRasGEF1B/miR-21-3p/MITA” network (51) and the “circSamd4a/miR-29a-3p/MITA” network (52) are two ceRNA networks associated with MITA in aquatic animals. Dual luciferase reporter assays have demonstrated that miR-21-3p and miR-29a-3p are available to bind circRasGEF1B and circSamd4a, respectively (**Figure 5B**). circRasGEF1B and circSamd4a acted as sponges, competitively binding to their respective target miRNAs (miR-21-3p and miR-29a-3p, respectively). In this process, the expression of circRasGEF1B, circSamd4a, and MITA (mRNA and protein) increased, while the expression of miR-29a-3p decreased, indicating a negative regulatory relationship between the above-mentioned circRNAs and miRNAs. The above-mentioned miRNAs were able to reduce the expression of MITA and inhibit the antiviral response. In contrast, those circRNAs could counteract the promotive effect of the above-mentioned miRNAs on the replication of SCRV, thereby maintaining the stability of the antiviral response and ensuring an appropriate inflammatory response. The present findings indicate that the “circRasGEF1B/miR-21-3p/MITA” network and the “circSamd4a/miR-29a-3p/MITA” network may participate in the activation of the NF-κB pathways and subsequent production of inflammatory factors after RNA virus infection in aquatic animals (51, 52).

Through the summary in this section, it can be seen that research on ceRNAs involved in the NF-κB pathways in aquatic animals is currently far from adequate, as only one fish species (miiuy croaker) has been relatively comprehensively studied, and thus more comprehensive and exhaustive efforts should be made in the future.

## 4 Concluding remarks

The immune regulatory function of ncRNAs in both vertebrates and invertebrates has been an intense research topic for more than 10 years. Although not as extensively or as thoroughly studied as in mammals, many ncRNAs have been identified as transcriptional regulators of key genes in both the canonical and non-canonical NF-κB pathways in aquatic animals, especially in teleost fishes. There is no doubt that continuous mining of ncRNAs with immune regulatory potential would benefit the sustainable development of the rapidly expanding aquaculture industry worldwide.

Despite the above-mentioned advances, we still need to note that (1) we are just beginning to understand the immune regulatory function of ncRNAs; like looking at a leopard through a tube, the range of aquatic species should be further expanded to clarify the differences in ncRNAs regulating NF-κB pathways between vertebrate and non-vertebrate aquatic animals; in addition, there is also much more research needed to extensively identify ncRNAs (especially lncRNAs, circRNAs, piRNAs, and novel ncRNAs) associated with the NF-κB pathways or other immune related pathways in aquatic animals (2); further exploration is necessary to clarify the explicit mechanisms concerning how “ncRNA-mRNA” axes or ceRNA networks regulate the NF-κB pathways or other immune related pathways in aquatic animals; and (3) given the important links between the NF-κB pathways and immune capability of aquatic animals, further studies on breeding-valuable ncRNA markers targeting genes of the NF-κB pathways will facilitate the development of more accurate and effective molecular-assisted breeding strategies in aquaculture.

## Author contributions

YYZ and YC conceived of the manuscript. The reference collection and data analysis were performed by TZ, YZ, and HY. The manuscript was written by YYZ, TZ, and YZ. All authors contributed to the article and approved the submitted version.
